# Three-dimensional heterostructure of metallic nanoparticles and carbon nanotubes as potential nanofiller

**DOI:** 10.1186/1556-276X-7-202

**Published:** 2012-03-29

**Authors:** Whi Dong Kim, Jun Young Huh, Ji Young Ahn, Jae Beom Lee, Dongyun Lee, Suck Won Hong, Soo Hyung Kim

**Affiliations:** 1 Department of Nanofusion Technology, College of Nanoscience and Nanotechnology, Pusan National University, 30 Jangjeon-dong, Geumjung-gu, Busan 609-735, South Korea; 2 Department of Nanomechatronics Engineering, College of Nanoscience and Nanotechnology, Pusan National University, 30 Jangjeon-dong, Geumjung-gu, Busan 609-735, South Korea; 3 Department of Nanomedical Engineering, College of Nanoscience and Nanotechnology, Pusan National University, 30 Jangjeon-dong, Geumjung-gu, Busan 609-735, South Korea; 4 Department of Nanofusion Engineering, College of Nanoscience and Nanotechnology, Pusan National University, 30 Jangjeon-dong, Geumjung-gu, Busan 609-735, South Korea; 5 Department of Nanomaterial Engineering, College of Nanoscience and Nanotechnology, Pusan National University, 30 Jangjeon-dong, Geumjung-gu, Busan 609-735, South Korea

**Keywords:** Metallic nanoparticles, Carbon nanotubes, Heterostructures, Polymer composites, Mechanical property

## Abstract

The effect of the dimensionality of metallic nanoparticle-and carbon nanotube-based fillers on the mechanical properties of an acrylonitrile butadiene styrene (ABS) polymer matrix was examined. ABS composite films, reinforced with low dimensional metallic nanoparticles (MNPs, 0-D) and carbon nanotubes (CNTs, 1-D) as nanofillers, were fabricated by a combination of wet phase inversion and hot pressing. The tensile strength and elongation of the ABS composite were increased by 39% and 6%, respectively, by adding a mixture of MNPs and CNTs with a total concentration of 2 wt%. However, the tensile strength and elongation of the ABS composite were found to be significantly increased by 62% and 55%, respectively, upon addition of 3-D heterostructures with a total concentration of 2 wt%. The 3-D heterostructures were composed of multiple CNTs grown radially on the surface of MNP cores, resembling a sea urchin. The mechanical properties of the ABS/3-D heterostructured nanofiller composite films were much improved compared to those of an ABS/mixture of 0-D and 1-D nanofillers composite films at various filler concentrations. This suggests that the 3-D heterostructure of the MNPs and CNTs plays a key role as a strong reinforcing agent in supporting the polymer matrix and simultaneously serves as a discrete force-transfer medium to transfer the loaded tension throughout the polymer matrix.

## Background

Nanostructured materials offer the advantages of a large specific surface area and strong mechanical properties. Thus, they have been used as fillers to reinforce polymer matrices. The interactions that occur at the molecular level owing to the large interfacial contact area between a polymer and nanofiller play a major role in dramatically enhancing the mechanical properties of hybrid polymer-nanofiller composites. Among the various characteristics of nanofillers, the dimensionality of nanofillers dispersed in polymer matrices has been of particular interest in recent years. Numerous research groups have examined the effect of nanofillers with dimensionalities of 0 or 1 on the mechanical properties of polymer composites. For example, the addition of small amounts of metallic nanoparticles (MNPs, 0-D) and carbon nanotubes (CNTs, 1-D) was found to significantly enhance the mechanical properties of polymer composites by as much as approximately 3% and approximately 100%, respectively [1-9]. We expect that MNPs added to a polymer matrix can only interact at several points along a particular polymer chain, whereas CNTs added to a polymer matrix can interact over the length of the polymer chain. Here, we posed the question of whether the combination of the 3-D heterostructures of both MNP-and CNT-based nanofillers in a polymer matrix can synergistically affect the mechanical properties of polymer-nanofiller composites. In order to answer this question, we systematically investigated the effect of nanofillers with the 3-D heterostructure of MNPs and CNTs on the mechanical properties of polymer composites. The heterostructure of MNPs and CNTs is composed of multiple CNTs grown radially on the entire surface of an MNP. These CNTs resemble a sea urchin and are hereafter referred to as sea urchin-like CNTs, or SU-CNTs [10,11]. In this study, an acrylonitrile butadiene styrene (ABS) was chosen as a specific example of polymer matrix because it is considered superior for its hardness, gloss, and toughness. We would intuitively expect that the SU-CNTs could have a novel enhancement effect on the mechanical properties of ABS polymer matrices due to their unique 3-D carbon nanostructure.

## Methods

**Figure 1:**
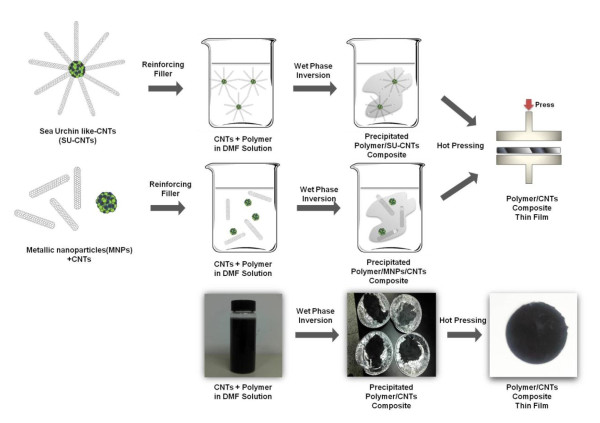
**Schematic diagram for the preparation of polymer-CNT composite thin films**.

The fabrication of 3-D SU-CNTs was previously described in detail [10,11]. Briefly, pure Ni-Al MNPs in the gas phase were first generated by conventional spray pyrolyzing reactor heated at 1,000°C under a flow of nitrogen (1,000 sccm) and hydrogen (100 sccm) gas. The particles were then continuously transported into a thermal chemical vapor deposition (CVD) reactor heated at 750°C where they were simultaneously mixed with controlled amounts of acetylene (10 sccm) and hydrogen (100 sccm) gas. This resulted in the growth of multiple CNTs on the entire surface of the in-flight Ni-Al MNPs, which were finally collected on a membrane filter. In order to prepare polymer-SU-CNT composite matrices, ABS was dissolved in dimethylformamide to a concentration of 15 wt% (Figure 1).

The SU-CNTs were then dispersed in the ABS solution at various concentrations, i.e., 0.25, 0.5, 1, and 2 wt%, by sonication (170 W, 40 kHz) for 3 h. The ABS/SU-CNT solution was mixed with filtered water to initiate the wet phase inversion process, in which a homogeneous mixture of ABS/SU-CNT was rapidly precipitated because of the solubility difference [12]. After the precipitated ABS/SU-CNT composites were dried at 80°C for 5 h, they were transformed into a thin film (diameter 58 mm; thickness 200 μm) using a hot press system (Model No. Mounting Press I, SSAUL BESTECH, Inc., Seoul, South Korea) at 180°C and 150 bar for 8 min. The ABS/SU-CNT composite thin film was then cut into specimens (40 mm × 6 mm × 200 μm) for tensile strength measurements (Model No. LRX Plus, Lloyd Inst. Ltd., Fareham, Hampshire, UK) following the standard tensile strength testing guidelines, ASTM D882 [13]. Seven tensile strength measurements were made for each sample, and the values were averaged to obtain the tensile strength of the fabricated ABS/SU-CNT composite. Here, the fabrication of ABS/MNP/CNT composite thin films and their tensile strength measurements were also made for comparison purpose. The morphologies of the SU-CNTs were characterized using a scanning electron microscope (SEM, S-4200, Hitachi, Chiyoda, Japan) operated at 15-20 kV and a transmission electron microscope (TEM, JEM-2100 F, Jeol Ltd., Tokyo, Japan) operated at 200 kV. The relative fraction of MNPs and CNTs in the prepared SU-CNTs was determined using thermogravimetric analysis (TGA) (TGA Q50, TA Inst., New Castle, DE, USA) with a heating rate of 10°C/min.

## Results and discussion

**Figure 2:**
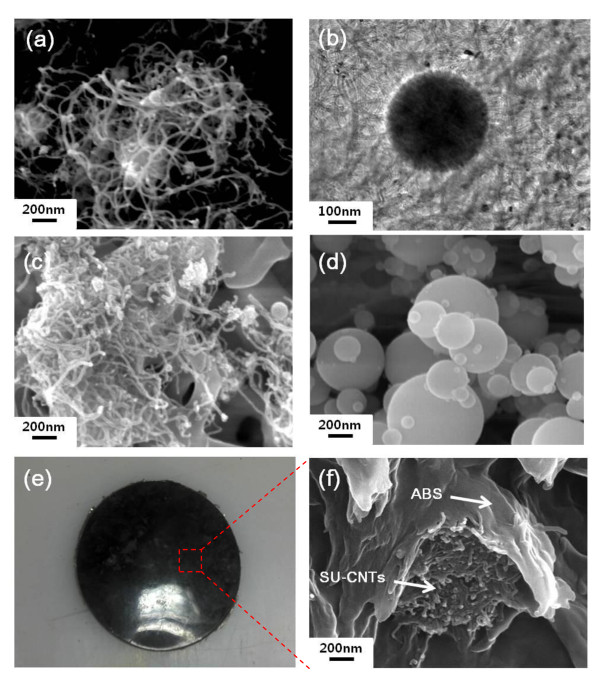
**SEM and TEM images**. (**a**) SEM and (**b**) TEM images of SU-CNTs, SEM images of (**c**) acid-treated SU-CNTs (i.e., metal core removed) and (**d**) Ni-Al bimetallic nanoparticles, and (**e**) photograph and (**f**) SEM image of ABS/SU-CNT composite thin film prepared by hot pressing.

Figure 2a and 2b show the 3-D SU-CNTs formed by the combination of spray pyrolysis and thermal CVD processes. It is clear that 1-D CNTs were grown radially on the entire surface of the 0-D MNP seeds. In order to examine the effect of nanofiller dimensionality on the mechanical property of the polymer composite, we needed to select low dimensional nanofillers with the same chemical compositions as the SU-CNTs for the purpose of comparison.

We first removed the MNP cores from the SU-CNTs using nitric acid treatment so that pure CNTs remained as shown in Figure 2c. We also separately prepared MNP cores using a spray pyrolysis process as shown in Figure 2d. We then added a mixture of MNPs and CNTs (MNPs/CNTs) or SU-CNTs to the polymer matrices as reinforcing agents to examine the effect of nanofiller dimensionality on the mechanical properties of polymer composites. Figure 2e shows an image of the ABS/SU-CNT composite thin film prepared by hot pressing, and Figure 2f shows an SEM image of the SU-CNTs dispersed in the ABS matrix.

**Figure 3:**
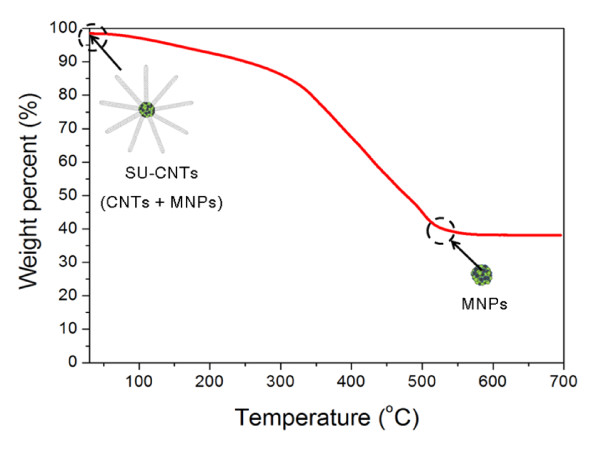
**Thermogravimetric analysis of SU-CNTs**.

In order to examine the ratio of MNPs to CNTs in the SU-CNT structure, we performed TGA of the SU-CNTs, the results of which are shown in Figure 3. The initial mass of the SU-CNTs decreased significantly as the temperature increased, due to the oxidation of the CNTs, and then remained at approximately 40% of the original sample mass at temperatures from 500°C-700°C. These results suggest that the SU-CNTs fabricated by this approach were composed of 40 wt% MNPs and 60 wt% CNTs. We prepared ABS polymer composite thin films containing (1) two different nanofillers, i.e., 0-D MNPs (40 wt%) and 1-D CNTs (60 wt%), and (2) a single nanofiller, i.e., 3-D SU-CNTs, by a combination of wet phase inversion and hot pressing to examine the effect of pure nanofiller dimensionality on the mechanical properties of the polymer composites.

**Figure 4:**
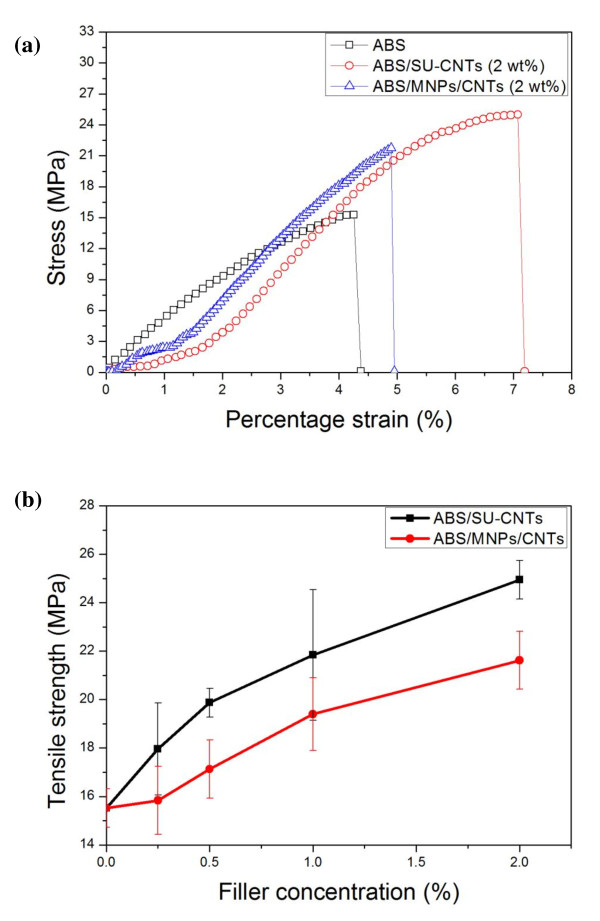
**Strain-stress curve and the evolution of tensile strength**. (a) Strain-stress curve of pure ABS, ABS/SU-CNTs (2 wt%), ABS/MNPs/CNTs (2 wt%), and (b) the evolution of tensile strength of ABS polymer composites with SU-CNTs or a mixture of MNPs/CNTs as a function of filler concentration.

Addition of the mixture of MNPs and CNTs or SU-CNTs as nanofillers clearly enhanced the mechanical properties of the ABS composite thin films, as shown in Figure 4a. Although the precise mechanism for the enhancement of mechanical properties of the ABS composite thin film is not entirely understood, molecular level interactions between CNT nanofillers and ABS matrices may play a major role. The improvement of tensile strength and elongation of ABS/MNP/CNT and ABS/SU-CNT composite compared to those of pure ABS is clearly related to the concentration and geometry of CNTs. The tensile strength and elongation of the ABS composite were increased by 39% and 6%, respectively, by the addition of the mixture of MNP/CNT nanofillers at a total mass concentration of 2 wt%. However, the tensile strength and elongation of the ABS composite thin film filled with 3-D SU-CNTs (2 wt%) were significantly increased by 62% and 55%, respectively. It should be noted that the severe aggregation of CNTs at higher nanofiller concentrations ≥ 2 wt% were observed, and it can cause relatively poor enhancement in mechanical properties of ABS/CNT composite thin films. Figure 4b shows the tensile strengths of the ABS polymer composites containing the SU-CNTs or the mixture of MNPs/CNTs as a function of nanofiller concentration. Note that the enhancement of the tensile strength of the ABS polymer with SU-CNTs was found to be much higher than that of the ABS polymer with MNPs/CNTs at various nanofiller concentrations. This suggests that the 3-D structure of the SU-CNTs is a key factor in its role as a strong reinforcing agent of the given ABS matrix. It was previously reported that the outer layers of the CNT filler could be easily removed from the inner layers due to weak coupling between CNT layers when tension was applied to the polymer-CNT composite [14]. However, unlike the typical 1-D CNTs, the 3-D SU-CNTs prepared by this approach had a unique structure in which the ends of multiple CNTs were strongly connected to the large surface of the MNP core so that migration of the outer layers of the CNTs was significantly suppressed. Another possible reason for the enhancement of the mechanical properties of the ABS/SU-CNT composite is that the loaded tension on the ABS/SU-CNT composite thin film was effectively distributed in the polymer matrix through the 3-D structure of the SU-CNT nanofillers. This is because the multiple CNTs grown radially on the surface of the spherical MNP core were unbundled and inherently well separated, allowing effective transfer of the loaded tension in every radial direction.

## Conclusions

We have examined the effect of nanofiller dimensionality on the mechanical properties of a polymer composite. We found that 3-D heterostructures of MNPs and CNTs (i.e., SU-CNTs) were much better reinforcing agents than the mixture of 0-D MNPs and 1-D CNTs and that they significantly improved the mechanical properties of the polymer composites. The unique structure of 3-D SU-CNTs prepared by this approach using a combination of spray pyrolysis and thermal CVD processes accounted for the enhancement of the mechanical properties of the polymer composites. The strong interaction of multiple CNTs with the large surface of the MNP core in the 3-D SU-CNT nanofillers facilitated strong coupling forces between the CNTs and polymer molecules. Simultaneously, the discrete CNTs grown radially on the entire surface of the MNP core allowed homogeneous transfer of the loaded tension throughout the polymer composite matrix.

## Competing interests

The authors declare that they have no competing interests.

## Authors' contributions

WDK and JYH contributed equally to this work as first co-authors. WDK and JYH made the synthesis and elemental analysis for CNT-reinforced polymer composites. JYA participated in the mechanical property measurements. JBL, DYL and SWH participated in the SEM and TGA measurements. SHK provided guidance to WDK and JYH as a supervisor and designed most of this project. All authors read and approve the final manuscript.
